# A systematic review of the application of computational grounded theory method in healthcare research

**DOI:** 10.1093/biomethods/bpaf088

**Published:** 2025-11-21

**Authors:** Ravi Shankar, Fiona Devi, Xu Qian

**Affiliations:** Clinical Research & Innovation Office, Tan Tock Seng Hospital, National Healthcare Group, Singapore, 308433, Singapore; Medical Affairs – Research Innovation & Enterprise, Alexandra Hospital, National University Health System, Singapore, 159964, Singapore; School of Civil, Aerospace and Design Engineering, University of Bristol, Bristol, BS8 1TR, United Kingdom

**Keywords:** computational grounded theory, natural language processing, digital health, topic modeling, qualitative methods

## Abstract

The integration of computational methods with traditional qualitative research has emerged as a transformative paradigm in healthcare research. Computational Grounded Theory (CGT) combines the interpretive depth of grounded theory with computational techniques including machine learning and natural language processing. This systematic review examines CGT application in healthcare research through analysis of eight studies demonstrating the method’s utility across diverse contexts. Following systematic search across five databases and PRISMA-aligned screening, eight papers applying CGT in healthcare were analyzed. Studies spanned COVID-19 risk perception, medical AI adoption, mental health interventions, diabetes management, women’s health technology, online health communities, and social welfare systems, employing computational techniques including Latent Dirichlet Allocation (LDA), sentiment analysis, word embeddings, and deep learning algorithms. Results demonstrate CGT’s capacity for analyzing large-scale textual data (100 000+ documents) while maintaining theoretical depth, with consistent reports of enhanced analytical capacity, latent pattern identification, and novel theoretical insights. However, challenges include technical complexity, interpretation validity, resource requirements, and need for interdisciplinary expertise. CGT represents a promising methodological innovation for healthcare research, particularly for understanding complex phenomena, patient experiences, and technology adoption, though the small sample size (8 of 892 screened articles) reflects its nascent application and limits generalizability. CGT represents a promising methodological innovation for healthcare research, particularly valuable for understanding complex healthcare phenomena, patient experiences, and technology adoption. The small sample size (8 of 892 screened articles) reflects CGT’s nascent application in healthcare, limiting generalizability. Future research should focus on standardizing methodological procedures, developing best practices, expanding applications, and addressing accessibility barriers.

## Introduction

Healthcare research increasingly confronts the challenge of analyzing vast amounts of unstructured textual data generated through electronic health records, social media platforms, patient forums, and digital health applications. Traditional qualitative research methods, while providing rich interpretive insights, often struggle with the scale and complexity of contemporary healthcare data. Simultaneously, purely computational approaches may lack the theoretical depth and contextual understanding essential for meaningful healthcare insights [1].

This gap is especially evident as healthcare systems globally generate unprecedented volumes of textual data containing valuable insights into health behaviors, treatment experiences, and system dynamics.

Computational Grounded Theory (CGT) emerges as a methodological bridge, integrating the interpretive rigor of grounded theory with computational techniques from machine learning and natural language processing. CGT represents a systematic approach to pattern detection, interpretive analysis, and theory development that leverages both human expertise and computational power [1]. The framework addresses a fundamental challenge in contemporary research: how to maintain the theoretical sensitivity and contextual understanding of qualitative methods while harnessing the analytical capacity of computational approaches to process large-scale data.

CGT differs from general NLP-assisted qualitative analysis through its three-phase approach: (i) computational pattern detection, (ii) iterative human interpretation within theoretical frameworks, and (iii) validation through additional analysis. Unlike purely computational approaches that may sacrifice interpretive depth, or traditional grounded theory limited by scale, CGT maintains theoretical sensitivity while leveraging algorithmic capacity for pattern recognition across datasets exceeding manual analysis capacity.

The healthcare domain presents unique challenges and opportunities for CGT application. Healthcare data encompasses diverse formats including clinical narratives, patient experiences, professional communications, and health-related social media content. These data sources contain valuable insights into health behaviors, treatment experiences, professional practices, and healthcare system dynamics. However, their volume and complexity often exceed the capacity of traditional qualitative analysis methods. For instance, a single healthcare forum may generate thousands of daily posts with nuanced perspectives on conditions, treatments, and care experiences—impossible to analyze manually.

Developing appropriate frameworks for analyzing healthcare text data extends beyond academic considerations. As healthcare systems globally face challenges such as aging populations, rising chronic disease burdens, and rapid technological change, the ability to derive timely insights from diverse data sources becomes critical for evidence-based policy and practice. CGT offers a methodological framework that can inform real-time decision-making while maintaining the depth of understanding necessary for addressing complex healthcare challenges.

This systematic review addresses following research questions: (i) How are CGT methods currently applied in healthcare research? (ii) What computational techniques and analytical procedures are employed? (ii) What theoretical and practical insights emerge from CGT applications? (iv) What are the advantages and limitations compared to traditional qualitative methods? (v) What recommendations emerge for future CGT research and practice?

## Methodology

This systematic review followed an adapted PRISMA framework suitable for methodological reviews, with modifications to accommodate the focus on methodological innovation rather than intervention effectiveness. This review protocol was registered in the International Prospective Register of Systematic Reviews (PROSPERO) with registration number CRD420251144413. The review process was designed to comprehensively analyze the application of CGT methods in healthcare research, examining both methodological approaches and substantive findings.

A comprehensive systematic search strategy aimed to identify all studies applying CGT methods in healthcare research contexts across multiple databases. The systematic search was conducted in May 2025 across five major electronic databases. These included PubMed/MEDLINE, Web of Science Core Collection, IEEE Xplore Digital Library, and ACM Digital Library. Additionally, Google Scholar was searched, with the first 200 results reviewed for each search string to ensure comprehensive coverage of relevant literature.

The included studies were selected based on predetermined criteria ensuring methodological rigor and relevance to healthcare contexts. Studies were required to demonstrate application of computational methods consistent with CGT principles, including pattern detection through computational techniques and interpretive refinement by human analysts. All studies needed to focus on healthcare-related research contexts, demonstrate clear integration of pattern detection and interpretive analysis, and be published in peer-reviewed venues to ensure quality.

The search strategy employed a combination of targeted search terms and defined eligibility criteria to identify relevant studies. Search terms included variations of CGT and related methodologies within healthcare contexts, such as: (“computational grounded theory” OR “computational GT”) AND (health* OR medical OR clinical OR patient*); (“topic modeling” OR “LDA” OR “latent dirichlet”) AND (“grounded theory”) AND (health* OR medical); and (“machine learning” AND “qualitative analysis”) AND (health* OR medical).

Studies were included if they applied CGT methods, were conducted in healthcare or medical research contexts, were published between till May 2025 (reflecting the period when CGT methodology was established), were peer-reviewed. Exclusion criteria were studies that used only traditional grounded theory methods, focused on non-healthcare domains, were limited to conference abstracts without full papers, or were systematic reviews or meta-analyses.

**Figure 1:**
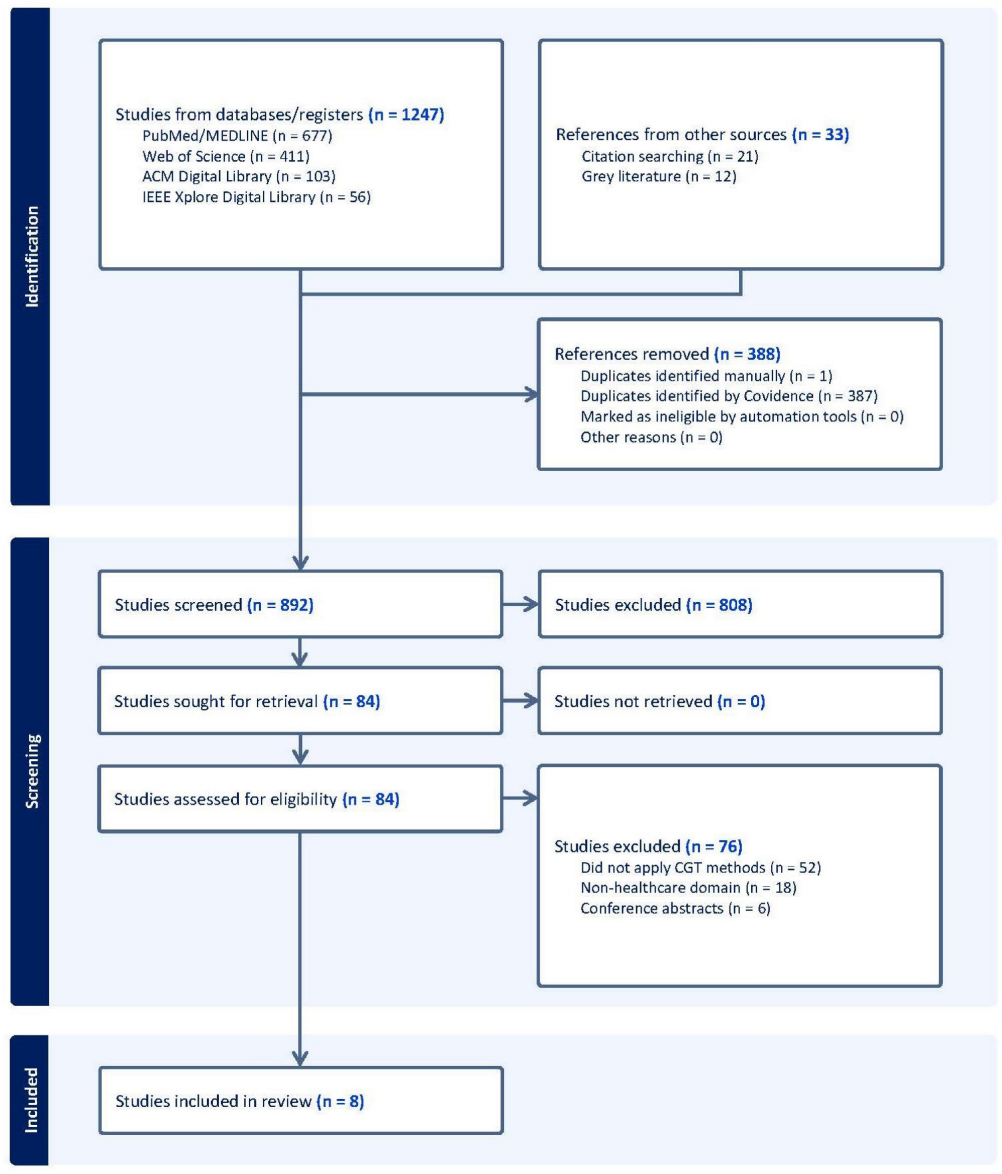
PRISMA flow diagram

The study selection process followed a systematic and transparent approach using Covidence systematic review software for both screening and data extraction. The initial database search yielded 1247 records. After removing duplicates, 892 unique records remained. These were screened by title and abstract, resulting in 84 potentially eligible articles. Full-text assessment of these 84 articles led to the final inclusion of 8 studies that met all inclusion criteria shown in the PRISMA flow diagram (Fig. 1). Two reviewers independently conducted the title and abstract screening, with any conflicts resolved through discussion. Full-text evaluations were also independently carried out by both reviewers, guided by the predetermined inclusion criteria. Inter-rater reliability for title/abstract screening achieved Cohen’s kappa = 0.89, indicating strong agreement. For full-text screening, kappa = 0.92. Disagreements were resolved through discussion until consensus.

A structured data extraction framework was developed to capture key methodological and substantive elements from each study. Study characteristics including authors, year of publication, healthcare domain, and primary research questions were systematically recorded. The methodological approach employed in each study was analyzed, focusing on computational techniques used, analytical procedures followed, and methods for integrating human interpretation with computational outputs. Data sources were characterized by type, volume, and characteristics of textual data analyzed. Key findings from each study were extracted, including substantive insights about healthcare phenomena, theoretical contributions to understanding healthcare issues, and methodological innovations in applying CGT. Finally, strengths and limitations of each study were assessed, particularly focusing on advantages over traditional methods and challenges encountered in implementation.

Given the methodological focus of this review, a narrative synthesis approach was employed to integrate findings across studies. This approach allowed for detailed examination of methodological variations, contextual factors, and emergent patterns in CGT application. The synthesis was organized into thematic categories addressing the review’s research objectives, enabling identification of common approaches, divergent strategies, and best practices across studies.

Quality assessment of the included studies was conducted using adapted criteria tailored for computational qualitative research. This evaluation focused on five key dimensions: methodological transparency and reproducibility; integration of computational and interpretive approaches; theoretical contribution and grounding; validity of computational procedures; and the practical applicability of findings. Each study was independently assessed and rated as high, moderate, or low quality across these dimensions to ensure a consistent and rigorous appraisal of methodological robustness and relevance.

## Results

### Overview of included studies

Following systematic screening, eight studies met inclusion criteria and were included in the final analysis. These studies demonstrate the breadth of CGT application across healthcare domains, methodological approaches, and geographical contexts (Table 1). Each study contributes unique insights into both the methodological development of CGT and its application to substantive healthcare challenges. A study explored COVID-19 risk conceptualizations in online communities using Reddit data, analyzing 500 000 comments to understand how different ideological communities construct understanding of pandemic risks [2]. Another study employed ChatGPT for qualitative coding assistance, representing an emerging but methodologically complex application [3]. Large language models introduce several validity concerns: (i) potential algorithmic bias embedded in training data, (ii) non-deterministic outputs requiring validation across multiple runs, (iii) risk of data leakage if sensitive healthcare information is processed, and (iv) limited transparency in decision-making processes (“black box” problem). The study addressed these limitations through systematic human validation of all AI-generated codes, maintaining researcher control over interpretive framework, and treating ChatGPT as an efficiency tool rather than authoritative analyst. Future CGT applications using LLMs must establish rigorous validation protocols and transparency standards. A study analyzed FemTech affordances through examination of period and fertility app reviews, identifying key features and user experiences that shape technology adoption [4].

**Table 1.** Summary of included studies.

Study	Healthcare domain	Data source	Sample size	Primary computational method	Key innovation
Adekunle *et al*. [2]	COVID-19 risk perception	Reddit (r/medicine)	500 000 comments	LDA topic modeling	Ideological community analysis
Yue *et al*. [3]	AI in qualitative research	Semi-structured interviews	31 interviews	ChatGPT-assisted coding	Human-AI collaboration framework
Guo *et al*. [4]	Women’s health technology	App reviews	21 489 reviews	LDA topic modeling	Affordance theory application
Vidyadharan *et al*. [5]	Diabetes management	Mixed qualitative data	Not specified	NLP & Deep learning	Semi-supervised learning approach
Figueroa *et al*. [6]	Health equity/racism	Medical literature	31 articles	Word embeddings	Racism narrative framework
Berriche *et al*. [7]	Health campaigns	Twitter	144 906 tweets	LDA + Sentiment analysis	Engagement segmentation
Weber *et al*. [8]	AI adoption	Reddit (medical subs)	181 threads	Clustering + thematic analysis	Mixed-method integration
Agudamu *et al*. [9]	Old-age welfare	Chinese literature	413 articles	Modified LDA	Policy theme identification

Additional studies expanded the application domains further. One investigation applied CGT to diabetes prevention and management using natural language processing techniques, demonstrating the potential for identifying evidence-based interventions from qualitative data [5]. Another line of work explored racism narratives in medical literature, developing a comprehensive framework for understanding how racial health inequities are discussed in academic publications [6]. Another study analyzed the #Movember health campaign through computational analysis of tweets, revealing patterns of engagement and awareness [7]. A separate analysis investigated medical professionals’ perceptions of AI using Reddit data, uncovering complex attitudes toward technology adoption [8]. Lastly, one project analyzed China’s old-age social welfare system through examination of academic literature, identifying key policy themes and recommendations [9].

### Computational techniques employed

The studies demonstrated diverse computational approaches within the CGT framework, with topic modeling emerging as the most commonly employed technique (Table 2). Six out of eight studies utilized Latent Dirichlet Allocation as their primary computational method for pattern detection. LDA enabled researchers to identify latent themes in large textual corpora without predetermined categories, allowing for discovery of unexpected patterns and relationships [10–14]. For instance, Adekunle, Foote [2] used LDA to identify 10 topics from 500 000 Reddit comments, revealing distinct conceptualizations of COVID-19 risk across ideologically different communities. The selection of optimal topic numbers varied across studies, with researchers employing various metrics including perplexity, coherence scores, and human judgment to determine appropriate granularity.

**Table 2.** Computational techniques and their applications.

Technique	Frequency	Studies using	Primary purpose	Advantages	Limitations
Latent Dirichlet Allocation (LDA)	6/8 studies	Adekunle, Guo, Berriche, Agudamu, Figueroa, Weber	Topic discovery	Unsupervised pattern detection	Requires topic number selection
Sentiment Analysis	3/8 studies	Berriche, Weber, Figueroa	Emotional valence	Captures affective dimensions	May miss context
Word Embeddings	2/8 studies	Figueroa, Vidyadharan	Semantic relationships	Context-aware representations	Computationally intensive
Deep Learning/NLP	2/8 studies	Vidyadharan, Yue	Complex pattern recognition	Handles non-linear patterns	Requires large datasets
Clustering	1/8 studies	Weber	Group identification	Identifies natural groupings	Sensitive to parameters
ChatGPT/LLM	1/8 studies	Yue	Coding assistance	Enhances efficiency	Requires validation

LDA coherence scores were used for topic number selection in [2], [4], and [7], following methods validated by Chang *et al*. [15] and Grimmer and Stewart [16]. However, topic modeling faces reproducibility challenges from random initialization and hyperparameter instability, addressed in reviewed studies through multiple runs with different seeds.

Sentiment analysis was incorporated in multiple studies to understand emotional dimensions of healthcare experiences. A study combined LDA with sentiment analysis to examine 144 906 tweets about the #Movember campaign, identifying both collective emotions and cognitive factors influencing health behavior engagement [7]. This combination of techniques enabled researchers to move beyond simple topic identification to understand the emotional valence associated with different health topics, providing insights into barriers and facilitators of health behavior change.

Advanced natural language processing techniques were employed to address specific analytical challenges. A study utilized deep learning approaches for analyzing qualitative data about diabetes management, demonstrating the potential for semi-supervised learning in healthcare contexts where labeled data may be limited [5]. Their approach combined traditional NLP techniques with neural network architectures to identify complex patterns in patient narratives about diabetes self-management. Another study exemplified sophisticated integration of multiple computational techniques, combining unsupervised clustering with qualitative thematic analysis to understand medical professionals’ AI perceptions [8]. This multi-method approach enabled identification of nuanced attitudes that might be missed by single-technique analyses.

Several studies employed novel computational approaches tailored to their specific research questions. Figueroa, Manalo-Pedro [6] developed a custom framework combining word embeddings with human coding to create a racism narrative typology, demonstrating how CGT can be adapted to address sensitive healthcare topics. Agudamu, Li [9] modified standard LDA procedures to include Jaccard similarity calculations for validating topic stability, introducing methodological innovations to ensure robustness of findings.

### Healthcare domains and applications

The studies spanned diverse healthcare contexts, demonstrating CGT’s versatility in addressing various health-related research questions. In public health and health promotion, three studies focused on population-level health issues. The COVID-19 risk perception study by Adekunle, Foote [2] identified associations between community membership and risk conceptualizations, revealing distinct thematic patterns across ideologically different communities. While topic modeling can identify co-occurrence patterns, it cannot establish causal relationships; the observed associations may reflect community identity shaping risk perception, self-selection of individuals into communities matching pre-existing views, or bidirectional influences. The #Movember analysis by Berriche, Crié [7] identified four segments of individual commitment to health campaigns, ranging from sympathizers to maintainers, each requiring different engagement strategies.

Digital health and technology adoption emerged as another major application area. Multiple studies examined how users perceive and adopt health technologies. Weber, Wyszynski [8] analyzed medical professionals’ perceptions of AI, identifying themes of job replacement anxiety and knowledge gaps that influence technology acceptance. Their findings revealed a complex relationship between AI knowledge and adoption intentions, moderated by fears about professional displacement. Guo, Liu [4] examined FemTech applications, revealing six primary affordances perceived by users: instrumental, engagement, esthetics, self-enhancement, community support, and partner support. These findings provide concrete guidance for technology developers seeking to create user-centered health applications.

Health equity and social justice applications demonstrated CGT’s capacity to address critical social issues in healthcare. Figueroa, Manalo-Pedro [6] applied CGT to examine racism narratives in medical literature, developing a framework for classifying narratives along an anti-racism spectrum from dismissal to actionable anti-racism. Their analysis revealed how medical literature perpetuates or challenges racist narratives through subtle linguistic choices and framing devices. This application shows how CGT can contribute to efforts to achieve health equity by making visible the narratives that shape medical knowledge and practice.

Clinical and health system applications illustrated CGT’s relevance to direct patient care and system improvement. Studies addressing clinical contexts included diabetes management [5] and the use of AI assistance in clinical practice [3]. The diabetes study identified key themes in patient self-management strategies, while the AI assistance study provided practical guidance for integrating computational support into clinical research. Agudamu, Li [9] demonstrated health system applications through their analysis of China’s old-age social welfare system, identifying seven key policy themes that could guide system reform.

### Data representativeness and ethical considerations

Digital data sources raise important limitations regarding population representativeness. Reddit and Twitter users skew younger, more educated, and technologically proficient than general patient populations, potentially missing perspectives of elderly patients, individuals with limited digital literacy, or marginalized communities with reduced internet access. App review data may over-represent extreme experiences (highly satisfied or dissatisfied users). These sampling biases limit generalizability of findings. Additionally, ethical considerations arise when analyzing social media data: while publicly posted, users may not anticipate research use; sensitive health discussions require careful privacy protection; and researchers must consider potential harms from reproducing stigmatizing narratives. Future CGT applications should explicitly address representativeness limitations and establish ethical frameworks for digital health data analysis.

### Methodological innovations and approaches

The reviewed studies demonstrated methodological approaches, including both faithful implementation of CGT framework and novel adaptations in applying CGT to healthcare research (Table 3). Most studies employed multi-phase analytical approaches consistent with CGT framework [1], typically involving three key stages. Pattern detection using computational techniques to identify initial patterns in the data served as the foundation, followed by pattern refinement through human interpretation to contextualize and validate computational findings. Finally, pattern confirmation through additional computational or qualitative analysis ensured robustness of results. This three-phase approach was adapted across studies to fit specific research contexts and questions.

**Table 3.** Methodological innovations in CGT application.

Innovation	Study	Description	Contribution to CGT
Three-phase iteration	All studies	Pattern detection → Refinement → Confirmation	Core CGT component (Nelson 2020), not novel
Human-AI collaboration	Yue *et al*. [3]	Structured integration of ChatGPT	Demonstrates AI augmentation potential
Mixed computational methods	Weber *et al*. [8]	Clustering + qualitative analysis	Shows complementary approaches
Theory-driven coding	Figueroa *et al*. [6]	Computational patterns + theoretical framework	Bridges induction and deduction
Validation metrics	Agudamu *et al*. [9]	Jaccard similarity for topic stability	Ensures reproducibility
Multi-level analysis	Adekunle *et al*. [2]	Community → Theme → Concept	Captures hierarchical patterns

Successful CGT applications demonstrated iterative collaboration between algorithmic pattern detection and expert knowledge, rather than replacing human interpretation with computational techniques. Yue, Liu [3] explicitly examined human-AI collaboration in qualitative analysis, finding that ChatGPT could enhance coding efficiency while requiring human oversight for depth and context. Their study provided detailed guidance on how researchers can effectively collaborate with AI systems, including strategies for prompt engineering, result validation, and maintaining interpretive control.

Studies leveraged diverse digital data sources previously inaccessible to traditional qualitative research, expanding the scope of healthcare research. Reddit discussions featured prominently in three studies, providing access to naturally occurring health-related discourse. Twitter data enabled real-time analysis of health campaign engagement. App reviews offered insights into user experiences with health technologies. Medical literature provided a corpus for analyzing professional discourse about health equity. Interview transcripts demonstrated CGT’s applicability to traditional qualitative data. This diversity of data sources illustrates CGT’s flexibility in addressing different types of healthcare research questions.

Several studies introduced novel analytical techniques that advance CGT methodology. Weber, Wyszynski [8] developed a mixed-method approach combining computational clustering with in-depth qualitative analysis, demonstrating how CGT can incorporate multiple analytical perspectives. Figueroa, Manalo-Pedro [6] created a custom coding framework that integrated computational pattern detection with theory-driven categorization, showing how CGT can be adapted for theory-testing as well as theory-generation. Agudamu, Li [9] introduced validation techniques using Jaccard similarity to ensure stability of topic models, addressing concerns about reproducibility in computational research.

### Validation and methodological rigor

While these studies demonstrate CGT’s potential, establishing validation standards remains critical. Key validation approaches employed included: (i) coherence metrics for topic model quality (LDA perplexity, coherence scores), (ii) inter-rater reliability for human coding phases (though only two studies reported kappa statistics), (iii) triangulation across multiple computational methods [8], and (iv) member checking or expert validation of interpretations (limited reporting). However, no consensus standards exist for validating computational-qualitative integration. Future work should establish reporting guidelines analogous to COREQ for traditional qualitative research, specifying minimum requirements for documenting computational procedures, validation approaches, and human-AI integration. Cross-disciplinary collaboration with fields like computational social science [17, 18] could inform these standards.

### Key findings and theoretical contributions

The studies generated significant theoretical insights through CGT application, demonstrating the method’s capacity for both discovery and theory development. Several studies developed new typologies or frameworks that advance understanding of healthcare phenomena. Adekunle, Foote [2] identified three key themes in COVID-19 risk perception: consequences of AI, physician-AI relationship, and proposed ways forward. These represent empirical findings from the included study rather than conclusions of this systematic review. Their analysis revealed how risk perceptions are shaped by community identity and ideological commitments rather than simply information processing. Figueroa, Manalo-Pedro [6] created a racism narrative framework with 4 broad categories and 12 granular modalities, providing a comprehensive system for analyzing how medical literature discusses racial health inequities. Guo, Liu [4] identified six FemTech affordances, offering a theoretical framework for understanding user engagement with women’s health technologies.

CGT enabled discovery of patterns not apparent through traditional analysis methods. Studies consistently reported identification of latent themes and relationships that would have been difficult to detect manually. Weber, Wyszynski [8] revealed the moderating role of AI knowledge on job replacement anxiety among medical professionals, a nuanced relationship that emerged through computational analysis of large-scale discourse. Berriche, Crié [7] identified a three-element structure of social movement commitment (segments, emotions, and cognitive factors) that provides a more complex understanding of health campaign engagement than previous models. These discoveries demonstrate CGT’s capacity to reveal hidden structures in healthcare discourse.

Studies also demonstrated how CGT can refine and extend existing theories. Several researchers used CGT findings to elaborate theoretical frameworks from other domains. The application of affordance theory in digital health contexts by Guo, Liu [4] extended understanding of how users perceive and engage with health technologies. The extension of risk perception theories to online communities by Adekunle, Foote [2] showed how traditional theories must be adapted for digital contexts. The development of anti-racism frameworks in medical discourse by Figueroa, Manalo-Pedro [6] provided new theoretical tools for understanding health equity. These theoretical contributions show how CGT can bridge empirical findings with abstract conceptualization.

It is important to note that none of the reviewed studies empirically tested the generalizability or external validity of their developed frameworks beyond the analyzed datasets. Figueroa *et al*.'s [6] racism narrative framework was applied to 31 articles; whether these 12 modalities comprehensively capture racism narratives across broader medical literature requires validation. Similarly, Guo *et al*.'s [4] six FemTech affordances emerged from period and fertility app reviews; their applicability to other digital health technologies remains unestablished. Future research should test whether CGT-derived frameworks transfer to new contexts, datasets, or populations.

### Advantages of CGT in healthcare research

The reviewed studies consistently identified several advantages of CGT over traditional qualitative methods (Table 4). Scalability emerged as a primary benefit, with CGT enabling analysis of datasets far exceeding traditional qualitative capacity. Studies analyzed hundreds of thousands of documents, with Adekunle *et al*. [2] examining 500 000 Reddit comments and Berriche *et al*. [7] analyzing 144 906 tweets. This scale of analysis would be impossible with manual coding methods, yet CGT maintained the interpretive depth characteristic of qualitative research. The ability to analyze large datasets is particularly valuable in healthcare research, where understanding population-level patterns while maintaining sensitivity to individual experiences is crucial.

**Table 4.** Advantages and challenges of CGT in healthcare research.

Aspect	Advantages	Challenges	Mitigation strategies
**Scale**	• Analyze 100 000 s of documents • Population-level insights • Comprehensive coverage	• 6/8 studies • Information overload • Difficulty prioritizing findings	• Hierarchical analysis • Human-guided filtering
**Pattern detection**	• Discover latent themes • Identify subtle relationships • Temporal trend analysis	• 7/8 studies • May miss context • Risk of spurious patterns	• Iterative validation • Domain expert review
**Reproducibility**	• Transparent procedures • Replicable analysis • Cumulative knowledge	• 5/8 studies • Parameter sensitivity • Platform dependencies	• Detailed documentation • Open-source tools
**Theory development**	• Novel frameworks • Cross-domain insights • Hypothesis generation	• 6/8 studies • Theory-data gap • Over-interpretation risk	• Strong theoretical grounding • Multiple analyst validation
**Resources**	• Efficiency at scale • Automated processing	• 8/8 studies • Technical expertise needed • Computational infrastructure	• Training programs • Collaborative teams

Pattern discovery capabilities of CGT revealed insights that human coders might miss. Computational techniques excel at identifying subtle patterns across large datasets, including latent topics in unstructured text that may not be immediately apparent to human readers, subtle linguistic patterns indicating bias or assumptions in medical discourse, and temporal trends in health-related discussions that emerge over extended time periods. These pattern discovery capabilities are particularly valuable for identifying emerging health concerns, tracking the evolution of health beliefs, and understanding how health information spreads through communities.

Enhanced reproducibility compared to purely interpretive methods addresses a key limitation of traditional qualitative research. Computational components of CGT can be documented and replicated, enabling other researchers to verify findings or apply similar methods to new datasets. Studies provided detailed documentation of computational procedures, including algorithm selection, parameter settings, and validation methods. This transparency enhances the credibility of qualitative research and enables cumulative knowledge building across studies.

The combination of computational pattern detection and human interpretation generated novel theoretical insights across all studies. CGT’s ability to process large amounts of data while maintaining theoretical sensitivity enabled researchers to identify patterns that inform theory development. Studies reported developing new conceptual frameworks, identifying previously unrecognized relationships between concepts, and generating hypotheses for future research. This theoretical innovation demonstrates CGT’s value not just for description but for advancing conceptual understanding of healthcare phenomena.

### Hierarchical importance of challenges

Based on study reporting frequency and methodological impact, challenges prioritize as follows: (i) Technical expertise requirements (reported by all 8 studies) represent the primary barrier to CGT adoption; (ii) Balancing computational scale with interpretive validity (7/8 studies) emerged as the central methodological tension; (iii) Reproducibility and validation standards (5/8 studies) require methodological development; (iv) Ethical and bias concerns (3/8 studies, but critically important) need greater attention despite limited reporting. The relatively low reporting of ethical challenges likely reflects underrecognition rather than absence of issues, particularly given documented biases in NLP algorithms and representativeness concerns with digital health data.

The relationship between methodological rigor and theoretical output quality provides insights into CGT best practices. Table 5 maps how different computational approaches and validation strategies contributed to the development of theoretical frameworks across studies. This mapping reveals that studies employing multiple iteration cycles and robust validation approaches tended to produce more comprehensive theoretical contributions.

**Table 5.** Mapping theoretical outputs to CGT methodological features.

Study	Theoretical output	Pattern detection method	Coding iteration cycles	Validation approach
Adekunle *et al*. [2]	Risk conceptualization themes	LDA (10 topics)	3 cycles	Expert review + community feedback
Figueroa *et al*. [6]	Racism narrative framework (4 categories, 12 modalities)	Word embeddings + manual coding	4 cycles	Multiple analyst validation
Guo *et al*. [4]	Six FemTech affordances	LDA + sentiment analysis	3 cycles	User experience validation
Weber *et al*. [8]	AI perception themes + moderating factors	Clustering + thematic analysis	Multiple cycles	Survey validation (*n* = 181)
Berriche *et al*. [7]	Three-element commitment structure	LDA + sentiment analysis	2–3 cycles	Statistical validation
Other studies	Domain-specific insights	Various NLP techniques	Variable	Limited reporting

The mapping in Table 5 reveals important patterns in CGT methodology. Studies employing multiple iteration cycles (3–4 cycles) and robust validation approaches (multiple analyst validation, survey validation, or statistical validation) produced more comprehensive theoretical frameworks. For instance, Figueroa *et al*. [6] combined 4 coding cycles with multiple analyst validation to develop a detailed racism narrative framework with 12 granular modalities. This suggests that methodological rigor—particularly iteration depth and validation stringency—directly influences theoretical output quality.

### Quality assessment

The quality assessment presented in Table 6 evaluates each study across five key dimensions, providing a systematic appraisal of methodological robustness and contribution to the field.

**Table 6.** Quality assessment summary.

Study	Methodological transparency	Computational-interpretive integration	Theoretical contribution	Computational validity	Practical applicability	Overall quality
Adekunle *et al*. [2]	High	High	High	High	Moderate	High
Yue *et al*. [3]	High	High	Moderate	Moderate	High	High
Guo *et al*. [4]	High	High	High	High	High	High
Vidyadharan *et al*. [5]	Moderate	Moderate	Moderate	Moderate	Moderate	Moderate
Figueroa *et al*. [6]	High	High	High	High	High	High
Berriche *et al*. [7]	High	High	Moderate	High	Moderate	High
Weber *et al*. [8]	High	High	High	High	High	High
Agudamu *et al*. [9]	Moderate	High	Moderate	High	Moderate	Moderate

Of the eight included studies, six were rated as high overall quality and two as moderate quality. No studies received low quality ratings.

### Challenges and limitations

Despite these advantages, studies identified several challenges in implementing CGT. Technical complexity emerged as a significant barrier, with successful CGT implementation requiring expertise in both computational methods and qualitative research. Researchers needed understanding of machine learning algorithms, programming skills for data processing and analysis, and ability to interpret and validate computational outputs. This technical requirement may limit accessibility for researchers without computational backgrounds, potentially creating disparities in who can conduct CGT research.

Balancing computational results with meaningful interpretation remains challenging. Several studies noted difficulties ensuring computational patterns translate to theoretically meaningful insights. The risk of being overwhelmed by computational outputs without adequate theoretical grounding was acknowledged across studies. Researchers emphasized the need for strong theoretical frameworks to guide interpretation of computational findings, highlighting that CGT is not simply about applying algorithms but about meaningful integration of computational and interpretive approaches.

Context loss represents another limitation, with some studies noting that computational techniques might miss contextual nuances captured by traditional qualitative methods. While computers excel at pattern detection across large datasets, they may miss subtle contextual factors that influence meaning, implicit cultural assumptions that shape discourse, and non-textual elements of communication such as tone or emphasis. Researchers addressed this limitation through careful human interpretation, but acknowledged that some contextual richness may be lost in computational processing.

Resource requirements for CGT implementation can be substantial. Beyond technical expertise, CGT often requires significant computational resources for processing large datasets, interdisciplinary collaboration between technical and domain experts, and time for iterative refinement between computational and interpretive phases. These resource requirements may limit CGT adoption in resource-constrained settings or for time-sensitive research questions.

## Discussion

This systematic review reveals that Computational Grounded Theory (CGT) represents a significant methodological advancement in healthcare research, successfully bridging computational analysis with interpretive depth. The eight reviewed studies demonstrate CGT’s versatility across diverse healthcare domains ie from COVID-19 risk perception to health technology adoption thereby indicating its broad applicability for analyzing complex health phenomena.

While CGT offers methodological advantages, important epistemic risks require acknowledgment. First, computational pattern detection may lead to reification—treating algorithmic outputs as objective reality rather than one analytical lens among many. Researchers must maintain critical distance from computational findings, recognizing that algorithms embed assumptions about language, meaning, and categorization. Second, researcher reflexivity, central to traditional grounded theory [19], faces challenges when computational processes mediate data interpretation. The researcher’s social position, theoretical commitments, and interpretive choices become less transparent when filtered through algorithms [17, 18]. Third, as data feminism scholars argue [20], computational methods risk amplifying existing power imbalances when applied to marginalized populations’ health narratives. CGT practitioners must actively resist these tendencies through critical reflection, transparent documentation of interpretive choices, and attention to whose voices computational methods amplify or silence.

The predominance of Latent Dirichlet Allocation (LDA) across studies (6/8) suggests its utility as a foundational technique for pattern detection in healthcare texts. However, the successful integration of diverse computational methods, including sentiment analysis, word embeddings, and deep learning demonstrates that technique selection should align with specific research questions. The evolution toward more sophisticated approaches, as seen in the AI perception studies, indicates a maturing methodological landscape.

Critical to CGT’s success is the iterative integration of human expertise with computational analysis. Rather than replacing human interpretation, the most effective applications employed cycles where computational techniques surfaced patterns for human interpretation, while domain expertise guided computational refinement. This synergy addresses longstanding tensions between qualitative depth and quantitative breadth in healthcare research.

The studies generated significant theoretical contributions, developing new frameworks for understanding technology affordances, racism narratives, and risk perception in digital contexts. CGT’s capacity to analyze large-scale data while maintaining theoretical sensitivity enabled insights impossible through either computational or qualitative methods alone. Practical applications emerged across intervention design, technology development, and policy formulation, demonstrating CGT’s value beyond academic inquiry.

This review has several limitations. First, the small number of included studies (8 of 892 screened articles, representing 0.9% of potentially relevant literature) reflects CGT’s nascent application in healthcare but substantially limits generalizability. The restrictive selection rate (84 articles to full-text review, 8 included) suggests either overly narrow inclusion criteria or genuine scarcity of rigorous CGT applications. Second, publication bias likely affects findings; studies with null results, implementation failures, or challenges may be under-represented. Third, English-language restriction excludes potentially relevant work in other languages. Fourth, quality assessment criteria required adaptation for this novel methodology, as standard qualitative (e.g. CASP) or quantitative (e.g. Cochrane) tools were insufficiently applicable. Fifth, narrative synthesis without meta-analysis limits quantitative comparison across studies.

## Conclusions

CGT represents a methodological development offering capabilities for analyzing large-scale textual data while maintaining theoretical depth. This review demonstrates applications across diverse healthcare domains, with studies reporting enhanced pattern discovery, theoretical insights, and practical applications compared to traditional methods. However, the small evidence base and methodological heterogeneity require cautious interpretation of CGT’s potential.

Key strengths emerging from reviewed studies include scalability enabling analysis of datasets exceeding traditional qualitative capacity, pattern discovery capabilities revealing latent themes, enhanced reproducibility through transparent computational procedures, and capacity for theoretical development through human-computer collaboration. CGT demonstrates particular value for analyzing rapidly evolving health contexts, diverse stakeholder perspectives, and large-scale patient experiences.

Future development should focus on standardizing methodological procedures, developing user-friendly tools to lower technical barriers, expanding applications to emerging healthcare challenges, and ensuring equitable access across research settings. Addressing current limitations requires investment in training programs, development of healthcare-specific computational tools, and establishment of ethical frameworks for large-scale health data analysis.

This review’s conclusions are constrained by the nascent state of CGT in healthcare, potential publication bias favoring successful applications, English-language restriction, and absence of long-term evidence regarding CGT’s impact on healthcare practice or policy. Claims of CGT’s “broad applicability” remain premature pending larger evidence base and formal quality comparisons with traditional methods. The field requires methodological maturation before definitive conclusions about CGT’s advantages can be drawn.

As healthcare systems generate increasing volumes of textual data, CGT provides a methodological framework potentially capable of transforming this information into meaningful insights while addressing current limitations through continued development. By integrating computational power with interpretive wisdom, CGT may offer an approach for healthcare research honoring both the complexity of human health experiences and opportunities presented by digital data. This systematic review provides a foundation for future CGT applications while highlighting the need for: (i) methodological standardization to enable comparison across studies, (ii) validation of CGT-derived insights through external replication, and (iii) critical attention to ethical, epistemological, and equity implications as the method evolves.

## Data Availability

The data supporting the findings of this systematic review are derived from publicly available published studies. All included studies are cited in the References section with complete publication details and DOIs where available. The systematic review protocol was registered in the International Prospective Register of Systematic Reviews (PROSPERO) with registration number CRD420251144413. The full search strategies, screening results, and data extraction forms used in this review are available from the corresponding author upon reasonable request. No new primary data were generated as part of this systematic review.
